# Supplementary material for: MetaRibo-Seq measures translation in microbiomes
Source: Nat Commun. 2020 Jun 29;11:3268. doi: 10.1038/s41467-020-17081-z (PMC7324362; doi:10.1038/s41467-020-17081-z)
Supplement: Supplementary file 10 — Supplementary Data 7 [file 41467_2020_17081_MOESM10_ESM.zip › File2/Confidence_High_Taxonomy/394897_out.krona.html]

Javascript must be enabled to view this page.

members
magnitude
magnitudeUnassigned
count
unassigned
taxon
rank

394897\_out

138

138
2
superkingdom

976
138
phylum

200643
138
class

order
138
171549

family
2
815

816
2
genus

46506
1
species

SRS050941\_contig\_number\_17601

species

SRS053398\_contig\_number\_437
47678
1

family
136
1853231

genus
136
283168

SRS011302\_contig\_number\_14665SRS013965\_contig\_number\_8255SRS014287\_contig\_number\_contig-100\_1180.96439SRS015960\_contig\_number\_13096SRS021948\_contig\_number\_23841SRS047044\_contig\_number\_16935SRS048060\_contig\_number\_3946SRS049712\_contig\_number\_18449SRS050422\_contig\_number\_15245SRS064276\_contig\_number\_29209SRS077552\_contig\_number\_3748SRS098073\_contig\_number\_11521SRS1041037\_contig\_number\_2283SRS143181\_contig\_number\_2404SRS143991\_contig\_number\_6227SRS144183\_contig\_number\_27754SRS148874\_contig\_number\_323SRS149325\_contig\_number\_10169SRS893369\_contig\_number\_7947
19


SRS045004\_contig\_number\_28140SRS101376\_contig\_number\_4713SRS143417\_contig\_number\_24567
species
1897010
3

species

SRS011084\_contig\_number\_28347SRS011134\_contig\_number\_25949SRS012849\_contig\_number\_25401SRS012969\_contig\_number\_25204SRS013098\_contig\_number\_29854SRS013476\_contig\_number\_35641SRS013521\_contig\_number\_contig-100\_1043.80671SRS013800\_contig\_number\_14696SRS014459\_contig\_number\_contig-100\_331.218310SRS015065\_contig\_number\_37856SRS015431\_contig\_number\_57646SRS015663\_contig\_number\_13264SRS015782\_contig\_number\_35588SRS015890\_contig\_number\_13768SRS016018\_contig\_number\_4374SRS016095\_contig\_number\_15932SRS016335\_contig\_number\_contig-100\_641.231731SRS016954\_contig\_number\_contig-100\_837.143474SRS017191\_contig\_number\_9547SRS017433\_contig\_number\_6509SRS018133\_contig\_number\_819SRS018836\_contig\_number\_17089SRS018888\_contig\_number\_8070SRS018984\_contig\_number\_8930SRS019161\_contig\_number\_24864SRS019397\_contig\_number\_5278SRS019445\_contig\_number\_16744SRS019601\_contig\_number\_12525SRS019787\_contig\_number\_4061SRS020233\_contig\_number\_47777SRS020271\_contig\_number\_219SRS020328\_contig\_number\_10598SRS020394\_contig\_number\_7952SRS021484\_contig\_number\_13388SRS022137\_contig\_number\_2460SRS022609\_contig\_number\_29470SRS023526\_contig\_number\_27003SRS024132\_contig\_number\_24636SRS024435\_contig\_number\_318SRS024492\_contig\_number\_18225SRS043001\_contig\_number\_11077SRS043701\_contig\_number\_12552SRS043768\_contig\_number\_25745SRS043841\_contig\_number\_981SRS044535\_contig\_number\_15281SRS045244\_contig\_number\_8222SRS046068\_contig\_number\_699SRS046369\_contig\_number\_10048SRS046717\_contig\_number\_9398SRS047014\_contig\_number\_28112SRS048164\_contig\_number\_16381SRS049446\_contig\_number\_10638SRS049773\_contig\_number\_25631SRS049995\_contig\_number\_18964SRS050026\_contig\_number\_11088SRS050925\_contig\_number\_28218SRS051031\_contig\_number\_24686SRS051610\_contig\_number\_8809SRS052697\_contig\_number\_27177SRS053214\_contig\_number\_13048SRS054352\_contig\_number\_21349SRS054905\_contig\_number\_15166SRS055982\_contig\_number\_3927SRS056259\_contig\_number\_17478SRS056273\_contig\_number\_21367SRS058770\_contig\_number\_18683SRS063190\_contig\_number\_10804SRS063985\_contig\_number\_24845SRS064557\_contig\_number\_12659SRS065504\_contig\_number\_23577SRS075773\_contig\_number\_contig-100\_217.202953SRS076804\_contig\_number\_20210SRS076976\_contig\_number\_10796SRS077127\_contig\_number\_10917SRS077194\_contig\_number\_26617SRS077231\_contig\_number\_24337SRS077730\_contig\_number\_10368SRS077849\_contig\_number\_9698SRS078176\_contig\_number\_11785SRS078242\_contig\_number\_27613SRS078665\_contig\_number\_4237SRS097958\_contig\_number\_10726SRS098827\_contig\_number\_2471SRS101433\_contig\_number\_9528SRS103987\_contig\_number\_26196SRS1041137\_contig\_number\_26117SRS104311\_contig\_number\_29446SRS104636\_contig\_number\_22528SRS104975\_contig\_number\_10307SRS1055083\_contig\_number\_3005SRS142503\_contig\_number\_40066SRS143598\_contig\_number\_13859SRS143876\_contig\_number\_23970SRS144506\_contig\_number\_43786SRS146764\_contig\_number\_28927SRS147139\_contig\_number\_22498SRS147346\_contig\_number\_35165SRS148091\_contig\_number\_12561SRS148159\_contig\_number\_40653SRS148196\_contig\_number\_18777SRS148424\_contig\_number\_24637SRS149075\_contig\_number\_173SRS149181\_contig\_number\_23669SRS892882\_contig\_number\_6932SRS893341\_contig\_number\_12887SRS893373\_contig\_number\_1424SRS971276\_contig\_number\_26718
28118
107


SRS018817\_contig\_number\_10932SRS077502\_contig\_number\_14859SRS078419\_contig\_number\_contig-100\_1232.203757SRS098881\_contig\_number\_15293SRS1041118\_contig\_number\_14421SRS1041129\_contig\_number\_5023
species
1637502
6

1263090
1
species

SRS016056\_contig\_number\_3319
